# Bilateral Interactions in the Mouse Dorsal Inferior Colliculus Enhance the Ipsilateral Neuronal Responses and Binaural Hearing

**DOI:** 10.3389/fphys.2022.854077

**Published:** 2022-04-19

**Authors:** Yun Liu, Yan Li, Yunyi Peng, Haipeng Yu, Zhongju Xiao

**Affiliations:** Department of Physiology, School of Basic Medical Sciences, Southern Medical University, Guangzhou, China

**Keywords:** auditory pathways, binaural enhance, inferior colliculus, ipsilateral response, synaptic current

## Abstract

The inferior colliculus (IC) is a critical centre for the binaural processing of auditory information. However, previous studies have mainly focused on the central nucleus of the inferior colliculus (ICC), and less is known about the dorsal nucleus of the inferior colliculus (ICD). Here, we first examined the characteristics of the neuronal responses in the mouse ICD and compared them with those in the inferior colliculus under binaural and monaural conditions using *in vivo* loose-patch recordings. ICD neurons exhibited stronger responses to ipsilateral sound stimulation and better binaural summation than those of ICC neurons, which indicated a role for the ICD in binaural hearing integration. According to the abundant interactions between bilateral ICDs detected using retrograde virus tracing, we further studied the effect of unilateral ICD silencing on the contralateral ICD. After lidocaine was applied, the responses of some ICD neurons (13/26), especially those to ipsilateral auditory stimuli, decreased. Using whole-cell recording and optogenetic methods, we investigated the underlying neuronal circuits and synaptic mechanisms of binaural auditory information processing in the ICD. The unilateral ICD provides both excitatory and inhibitory projections to the opposite ICD, and the advantaged excitatory inputs may be responsible for the enhanced ipsilateral responses and binaural summation of ICD neurons. Based on these results, the contralateral ICD might modulate the ipsilateral responses of the neurons and binaural hearing.

## Introduction

Mammals have two ears. With two ears, humans and animals can immediately determine the location of noise produced by something without having to adjust their heads (Lynne et al., 2012; Micheal et al., 2018). Binaural hearing benefits spatial perception, auditory scene analysis and listening comprehension (He et al., 2017; Snapp and Ausili, 2020; Gallun, 2021). The auditory ascending system has many hierarchical partitions of nuclei in the mammalian brain from the cochlear nuclei (CN) to the auditory cortex (Winer and Schreiner, 2005). From the CN, most fibres cross the midline and ascend to the contralateral superior olivary complex (SOC) or to the contralateral nucleus of the lateral lemniscus nuclei (NLL) and then to the contralateral inferior colliculus (IC). The IC is the ultimate end point of many of the brainstem nuclei outputs (Ono and Ito, 2015). These brainstem nuclei include regions that detect the temporal difference between sounds reaching each ear or the difference in sound intensity between ears and hence the localization of the angle from which the sound is derived. Neurons in the inferior colliculus receive direct or indirect, monaural or binaural, ipsilateral or contralateral, and inhibitory or excitatory projections from many auditory nuclei, providing an anatomical and physiological basis for the binaural integration of information by the inferior colliculus.

Generally, the IC contains different subdivisions in which the central nucleus of the inferior colliculus (ICC) participates in the lemniscus pathway and is the terminating point of the most subcollicular ascending fibres. The dorsal nucleus of the inferior colliculus (ICD) receives inputs from the ventral CN rather than the dorsal CN and is the main region responsible for descending feedback information (Caird and Klinke, 1987; Gooler et al., 1996; Nakamoto et al., 2015). Recently, a growing number of studies have focused on ICD and deviated from this idea of simplistic subdivisions that process non-overlapping information (Barnstedt et al., 2015; Chen and Song, 2019; Wong and Borst, 2019). These studies are primarily concerned with investigating the processing of complex acoustical information and multimodal information, as well as experience-dependent plasticity based on the downstream feedback from the auditory cortex (Geis and Borst, 2013a; Stebbings et al., 2014; Wong and Borst, 2019; Sun et al., 2020; Oberle et al., 2022). Binaural processing or binaural properties are less frequently detected in the ICDs. However, a recent report documented that ascending inputs also predominated in the ICD (Chen et al., 2018). Thus, binaural processing within the ascending inputs may also be important in the ICD. Previous studies on IC binaural information computation focused on how neurons process sound localization cues, including binaural time and sound level differences (Lu and Jen, 2003; Grana et al., 2017). Although space-specific neurons have been identified in different animals, only a small proportion of the neurons shows selective sensitivity, and these neurons are probably only responsible for the encoding of sound source localization. As the ICD also receives bilateral brainstem nuclei projections as topographically organized inputs, binaural integration may not be exclusively performed in the ICC. In addition, ICC neurons show contralateral domination, and selectivity of contralateral or ipsilateral inputs instead of summation (Xiong et al., 2013; Wei et al., 2018). Binaural information integration appears to be complete before projection onto the ICC rather than completed within the ICC, and the function of ICC neurons is to further strengthen low central processing results (Wei et al., 2018; Wu et al., 2021). An interesting question is whether the binaural responses in the ICD are similar to those in the ICC. Although some papers indicated that neurons in the ICD show different spike latencies and frequency tuning properties from those of neurons in the ICC, their research was limited to sound delivery (Syka et al., 2000; Lumani and Zhang, 2010). The difference in the properties is presumed to arise from the different local interactions in the IC. Because the thalamus lacks functional interconnection, the IC commissure is the last place where auditory information can interact across opposite sides until it reaches the cortex (Malmierca, 2003; Cai et al., 2019). Neuronal responses in the inferior colliculus may be modulated by intercollicular commissural projections (Malmierca et al., 2005). Researchers have not determined how intercollicular commissures contribute to the binaural responses of ICD neurons.

In this study, we compared the differences in binaural properties between the ICD and ICC using loose-patch recordings under binaural, ipsilateral and contralateral stimulation. We found discrepancies in the binaural responses of the ICD and, specifically, lower thresholds and stronger responses to ipsilateral stimuli. Combining pharmacological injections and electrophysiological recordings, we confirmed that the contralateral ICD provides excitatory inputs to support the responses to ipsilateral stimuli. Our results will improve the understanding of the processing of binaural information in the auditory system.

## Materials and Methods

### Animals

Seventy-nine female C57BL/6 mice (4–6 weeks old, 14–18 g) were used for this study in total. All mice were supplied by the Experimental Animal Center of Southern Medical University and housed in a standard exhaust ventilated laboratory animal cage with a regular light/dark cycle and unlimited food and drink. All animal experimental protocols in our study were supervised by the Animal Care and Use Committee of Southern Medical University.

### Animal Surgery

The surgical procedures used for mouse electrophysiological recordings were similar to those methods that we previously reported (Wei et al., 2018; Liu et al., 2019; Wang et al., 2019; Wu et al., 2021). Mice were anaesthetized with pentobarbital (60–80 mg/kg, i. p., Sigma, United States) and atropine (0.25 mg/kg, s. c., Nandao, China). When the pedal withdrawal reflex of the animal vanished, its hair and the skin on its head were snipped off and a customized nail was fastened to the skull with adhesive. Next, the skull was opened without removing the dura by generating a small perforation (0.5 × 0.5 mm^2^) above the right ICD or ICC (−5.0 or−5.2 mm from bregma, one or 0.5 mm lateral to the midline) according to the coordinates of the Mouse Brain Atlas (Paxinos and Franklin, 2001). Vaseline was applied to the exposed dura, and lidocaine hydrochloride (Suicheng, China) was applied to the incision as an anaesthetic. The mouse was brought back to its own cage after surgery for recuperation and was given antibiotic ointment once a day to prevent infection.

### *In vivo* Loose-Patch and Whole-Cell Recordings

Three to 4 days after surgery, the mouse was re-anaesthetized with urethane (Sigma, United States) at a dose of 0.8–1 g/kg as a 20% solution via intraperitoneal injection for recordings. The mouse’s head was fixed by screwing the affixed nail into a metal pole. Following the removal of the Vaseline and dura, a glass micropipette (tip diameter: 1.5 μm, resistance: 6–7 MΩ) was used to probe the ICD region with a micromanipulator (Siskiyou Inc., United States). A 700B amplifier (Axon, United States) was used to take our electrophysiological recordings. To perform *in vivo* loose-patch recordings, the micropipette containing artificial cerebrospinal fluid (ACSF; in mM: 124 NaCl, 2.5 KCl, 25 NaHCO_3_, two CaCl_2_, one MgCl_2_, 1.23 NaH_2_PO_4_, 20 glucose, pH = 7.28) and 0.5% biocytin was attached to the neuron with a loose seal (0.2–1 MΩ) (Liu et al., 2019; Wu et al., 2021). The spike shapes (action potentials) were monitored and stored by Brain Ware (TDT 3, Tucker-Davis Technologies, United States) during recording. The data of neurons with consistent spike shapes would be adopted. The pipette solution containing the action potential inhibitor formula (in mM: 125 Cs-gluconate, five TEA-Cl, four MgATP, 0.3 GTP, eight phosphocreatine, 10 HEPES, 10 EGTA, two CsCl, and one QX-314, pH = 7.25) was used in whole-cell recordings and was the same as that described in our previous research (Liu et al., 2019). To achieve the whole-cell configuration, a negative pressure was applied to break the cell membrane when the pipette patched a neuron with a giga-ohm seal. The membrane capacitance and series resistance were compensated after a break-in. The series resistance (20–40 MΩ) was compensated for 50–60%. Signals were filtered at 5 kHz and sampled at 10 kHz. Only neurons with resting membrane potentials lower than−55 mV and a stable series resistance were used for further analysis (Wei et al., 2018; Liu et al., 2019; Wu et al., 2021).

### Sound Signal Generation

The sound stimulation programme was performed as described in our previous paper (Wei et al., 2018). Tone bursts of 50 m duration with 5 m rise/fall timings were presented by the Tucker-Davis Technologies System 3 (TDT 3, Tucker-Davis Technologies, United States) and Brain Ware software in a randomized sequence (2–64 kHz, at 0.1 octave interval, 0–70 dB, in 10 dB step). Two closed-field loudspeakers (EC1, 2–110 kHz) transmitted sound to the ears of the mouse via silicone tubes. Each silicone tube was placed into the external ear canal to minimize the crosstalk. The loudspeakers were calibrated with a 1/4-inch microphone (Brüel and Kjaer 4,135, Naerum, Denmark) and a measurement amplifier (Brüel and Kjaer 2,610, Naerum, Denmark) regularly to ensure a flat frequency response. During speaker calibration, the microphone was placed close to the speaker, and the voltage values of each pure tone signal were adjusted according to the indication of the calibrator that controlled the sound level to match at 70 dB.

### Microinjection of Lidocaine

A local injection of lidocaine (containing 0.5% biotinylated dextran amines) was administered to inactivate the contralateral ICD. A small craniotomy window was generated over the contralateral ICD opposite to the recording site. A microsyringe (UWC4, World Precision Instruments, United States) was used to deliver lidocaine via a glass micropipette with a bevelled opening tip (20–30 μm). The injection site was approximately 250 μm lower than the surface of the brain. The drugs, totalling 100 nL, were administered at a speed of 0.2 μL/min. In some recordings, loose-patch recordings were performed in the ICD of the injection site before experiments to verify the effect of lidocaine.

### Virus Injection

Coordinates for injections were the same as those used for recording. The injection site for ICD or ICC was 250 μm or 1,000 μm lower than the surface of the brain. Mice were anaesthetized with 1.5% isoflurane. A 0.2 × 0.2 mm^2^ craniotomy window was made through a tiny incision in the skin overlying the ICD and ICC. In the retrovirus experiments, rAAV-CAG-mCherry-WPRE-pA (retro) and rAAV-CAG-EGFP-WPRE-pA (retro) were used. In the optogenetic experiments, rAAV-CaMKIIα-ChR2-mCherry and rAAV-CaMKIIα-eNpRH-EYFP (BrainVTA, Wuhan, China) were used. A bevelled glass micropipette with a tip opening diameter of approximately 30 μm was used to transport the virus, which was subsequently coupled to a microsyringe pump (KDS310, KD Scientific, United States). The injection rate was set at 10 nL/min, with a viral solution volume of 40 nL for tracing experiments and 60 nL for optogenetic experiments. The pipette was left in place for 5 min before being progressively lifted out. Then, the wound was stitched up and antibiotic ointment was applied before the mice were returned to their cages. Mice were given at least 3 weeks to recuperate.

### Optogenetic Activation

To activate the light-sensitive channel expressed in virus-infected neurons, an optic fibre (200 μm, NA: 0.22, Newdoon, China) linked to a laser instrument (470 and 590 nm, Thorlabs, United States) was placed on the surface of the IC. The laser instrument was connected to the TDT3 system and controlled by Brain Ware software. In these experiments, the connection of the optic fibre and the eyes of mouse were shaded with black tape to prevent light leakage.

### Histological and Image Processing

After the experiments, mice were deeply anaesthetized by administering an excess of pentobarbital (120 mg/kg, i. p.). Then, we transcardially infused 0.1 M phosphate-buffered saline and 4% paraformaldehyde (PFA). Their brains were dissected out and postfixed in 4% PFA for 12 h before being sliced coronally into 100 µm thick slices using a freezing microtome (CM1905, Leica, Germany). For biocytin staining, slices were permeated with 0.3% Triton for 2 h and then incubated with streptavidin-Cy3 (1:200, Molecular Probes, United States) for 4 h. 4′,6-diamidino-2-phenylindole dihydrochloride (DAPI, 0.25 μg/ml) was used for nuclear counterstaining. All slices were analysed by a confocal system (A1Rs, Nikon, Japan). To determine the borders of brain structures, the imaged tissues were compared to the Mouse Brain Atlas (Paxinos and Franklin, 2001).

### Data Analysis

The spike tonal receptive field (TRF) was measured at least three times, and the synaptic current TRF was measured one to two times. The count of spikes was calculated from a 0–150 m time window in the poststimulus spike time histogram. The values of spike and synaptic TRFs were calculated automatically by our customized program in MATLAB 2016a. The characteristic frequency (CF) was chosen as the frequency with the strongest response at the threshold. The best frequency (BF) was chosen as the largest response frequency overall at all intensities. The spike response latency was calculated as the average of the first evoked spike in each repetition.

### Statistics

Two-sample or paired *t*-tests were used to compare two groups. One-sample *t*-tests were used to test whether an index of samples followed zero. For nonnormally distributed data, the Mann–Whitney test was applied to evaluate significance. Two-sample Kolmogorov–Smirnov tests were used to test the distribution of the two datasets. Two-way ANOVA was used for datasets with two categorical variables. A significance level of *p* < 0.05 was used. Data were fit and plotted using Origin software (version 8).

## Results

### ICD Neurons Have Stronger Responses to Ipsilateral Stimuli Than Those of ICC Neurons

**FIGURE 1:**
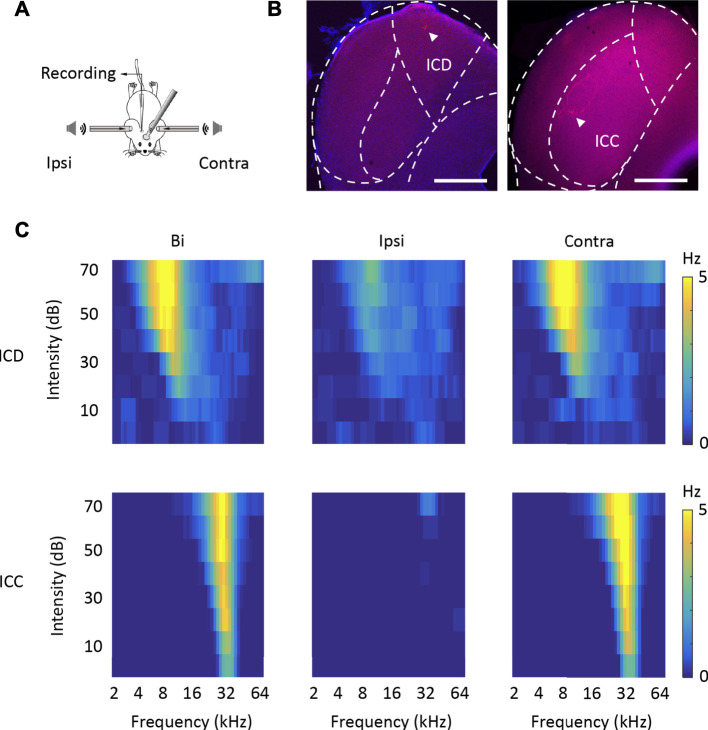
Loose-patch recording of ICD and ICC neurons in anaesthetized mice. **(A)** Schematic diagram of the electrophysiological recording model. **(B)** Two representative biocytin-labelled neurons in red show the locations of recorded ICD (left panel) and ICC (right panel) neurons. The borders of IC subdivisions are manually outlined based on the mouse brain atlas by Paxinos and Franklin (2001) at bregma −4.96 mm (left panel) and−5.02 mm (right panel). Scale bar, 500 μm. **(C)** The tone receptive fields of recorded ICD (upper panels) and ICC (lower panels) neurons in response to binaural (left panels), ipsilateral (middle panels), and contralateral (right panels) tone stimuli.

**FIGURE 2:**
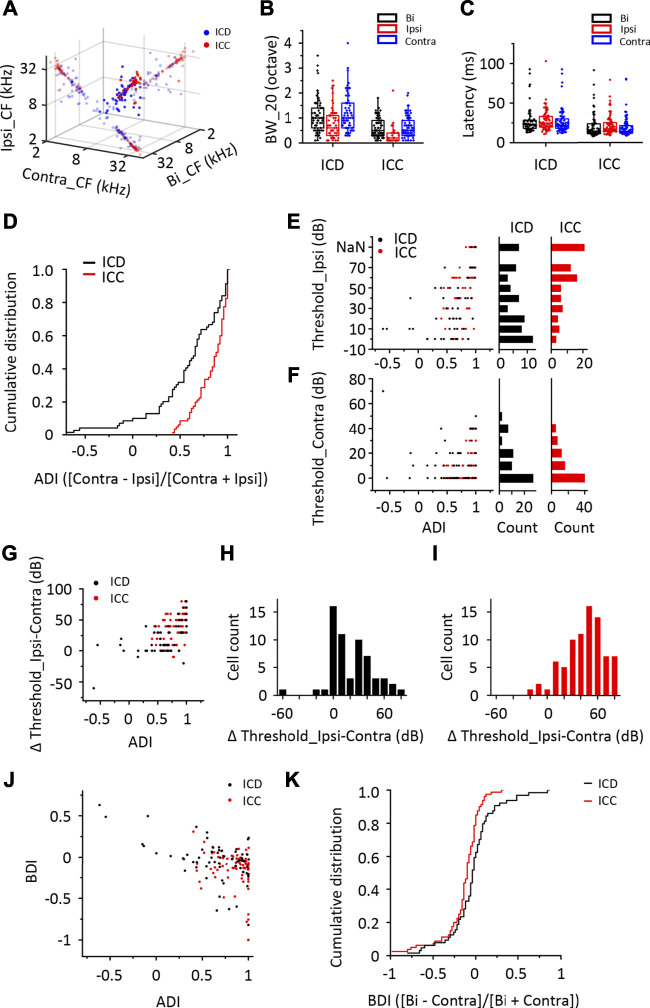
The response of ICD neurons to ipsilateral stimuli differs from that of ICC neurons. **(A)** Relationship of the characteristic frequency (CF) of ICD and ICC neurons exposed to binaural, ipsilateral, and contralateral stimuli. The small nontransparent points show the projection of 3D points in the 2D coordinate. **(B)** The population of bandwidth of responses to binaural, ipsilateral, and contralateral stimuli above a minimum threshold of 20 dB (BW_20) in recorded ICD and ICC neurons. **(C)** The population of first spike latency in recorded ICD and ICC neurons. **(D)** Cumulative distributions of the aural dominance index (ADI) in the ICD and ICC. Cells with an ADI = 1 exhibit only contralaterally evoked spike responses. **(E,F)** The ADIs of ICD and ICC neurons versus the minimum thresholds of ipsilateral **(E)** and contralateral **(F)** tonal receptive fields (TRFs). The distributions of the minimum thresholds of ipsilateral **(E)** and contralateral **(F)** TRFs in ICD and ICC neurons. NaN indicates that the threshold is out of detection range. **(G)** The ADIs of ICD and ICC neurons versus the threshold difference between monaural TRFs (ipsilateral-contralateral). **(H–I)** The distributions of the difference in thresholds between monaural TRFs (ipsilateral-contralateral) in the ICD **(H)** and ICC **(I)**. **(J)** The ADIs of ICD and ICC neurons versus the binaural dominance index (BDI). Cells with a BDI >0 exhibit enhanced binaural responses. **(K)** Cumulative distributions of the BDI in the ICD and ICC. The box-plots show quartiles and outliers.

First, using *in vivo* loose-patched recording, we recorded the binaural and monaural spike TRFs from neurons in two different IC subregions, the ICD and ICC, in anaesthetized mice. Sixty-nine ICD and 80 ICC neurons were recorded from 34 mice in this experiment (Supplementary Figure S1). The recording model is shown in Figures 1A, B. shows representative cells labelled with biocytin in the ICD and ICC regions, which indicates the reliability of the recording site. Figure 1C shows the binaural and monaural (ipsilateral and contralateral) spike TRFs in two neurons. Both neurons exhibited a nice receptive response to binaural and contralateral stimuli with a low minimum threshold at 0 dB. The ICD neuron (Figure 1C, upper panel) had a broader receptive field, whereas the ICC neuron (Figure 1C, lower panel) had a narrow receptive field that included only a few frequencies near the CF. Although their ipsilateral tone-evoked spikes were lower than those of binaural and contralateral spikes, the ipsilateral response of the ICD neuron was significantly higher, and the area of receptive fields was roughly matched to those of binaural and contralateral spikes along with their CFs. The ICC neuron responded to ipsilateral tones at higher sound intensity. We systemically analysed the data to compare this difference in the ipsilateral responses of the ICD and ICC. Most of the ICD and ICC neurons exhibited the same CF following exposure to contralateral and binaural stimuli, while the ICC matched well (Figure 2A). The summary of BW_20, which represents bandwidths of TRF, indicated that ICD neurons had a broader receptive field (Figure 2B, ICD: mean: bi, 1.12 octave; ipsi, 0.79 octave; contra, 1.93 octave; median: bi, 1 octave; ipsi, 0.65 octave; contra, 1 octave; ICC: mean: bi, 0.6 octave; ipsi, 0.35 octave; contra, 0.65 octave; median: bi, 0.5 octave; ipsi, 0.2 octave; contra, 0.5 octave; two-way ANOVA, *F*_1,365_ (ICD vs. ICC) = 66.86, *p* < 0.0001). Meanwhile, ICD neurons had longer latencies than those of ICC neurons (Figure 2C, ICD: mean: bi, 27.70 m; ipsi, 28.21 m; contra, 27.46 m; median: bi, 23.06 m; ipsi, 24.71 m; contra, 22.07 m; ICC: mean: bi, 20.34 m; ipsi, 22.26 m; contra, 19.21 m; median: bi, 14.41 m; ipsi, 17.38 m; contra, 14.23 m; two-way ANOVA, *F*_1,416_ (ICD vs. ICC) = 25.314, *p* < 0.0001). To compare responses to contralateral and ipsilateral stimuli quantitatively, an aural dominance index (ADI) was calculated by dividing the difference between contralateral and ipsilateral responses across the whole TRF by their sum ([Contra − Ipsi]/[Contra + Ipsi]). The distributions of ADI for ICD and ICC neurons were different (Figure 2D, Kolmogorov–Smirnov test, *p* < 0.0001). The ICC contained more neurons with ADIs near one (40/80, ADI >0.9), while the ICD had fewer (19/69) and even contained some individual neurons with ADIs less than zero (7/69). Furthermore, we compared the minimum thresholds of ipsilateral and contralateral TRFs and found that the ICD had a lower response threshold to ipsilateral stimuli (Figure 2E, ICD: mean, 34.64 dB, median, 20 dB, ICC: mean, 57.13 dB, median, 60 dB, Mann–Whitney test, *p* < 0.0001), but no significant difference was observed under the contralateral condition (Figure 2F, ICD: mean, 10.13 dB, median, 10 dB, ICC: mean, 14.06 dB, median, 5 dB, Mann–Whitney test, *p* = 0.287). Moreover, the differences between ipsilateral and contralateral thresholds were distinguished in the ICD and ICC (Figures 2G–I, when the neuron’s MT was greater than 70 dB, we set it to 80 dB). The differences between ipsilateral and contralateral thresholds in the ICC were larger than those in the ICD (Figures 2H, I, ICD: mean, 19.28 dB, median, 10 dB, ICC: mean, 44.38 dB, median, 50 dB, Mann–Whitney test, *p* < 0.0001). Moreover, we calculated a binaural dominance index (BDI) that measures the difference between binaural and contralateral responses across the whole TRF divided by their sum ([Bi − Contra]/[Bi + Contra]) to compare the responses between the binaural and contralateral stimuli. The distributions of the BDI for ICD and ICC neurons were different (Figures 2J, K, Kolmogorov–Smirnov test, *p* = 0.012). When comparing the two datasets of ICD and ICC, the ICC had more neurons (63/80) with a BDI less than zero, while more neurons (27/69) in the ICD exhibited a BDI greater than zero. Based on this result, more ICD neurons displayed greater binaural responses than they displayed monaural responses, suggesting that the neurons sum the contralateral and ipsilateral responses together under binaural stimulation. In addition, the ADI and BDI points of the ICC and ICD neurons were negatively correlated (Figure 2J, Pearson correlation, ICC: R = −0.279, *p* = 0.012, ICD: R = −0.611, *p* < 0.0001). In summary, we compared the binaural and monaural spike TRFs of ICD and ICC neurons and found that the responses to ipsilateral stimuli were different between these two datasets. A much higher proportion of ICD neurons than ICC neurons responded to ipsilateral stimuli and showed a high spike rate and low threshold that were the same as their responses to contralateral stimuli.

### The Difference Between the ICD and ICC Pathways in Transferring the Bilateral Information

**FIGURE 3:**
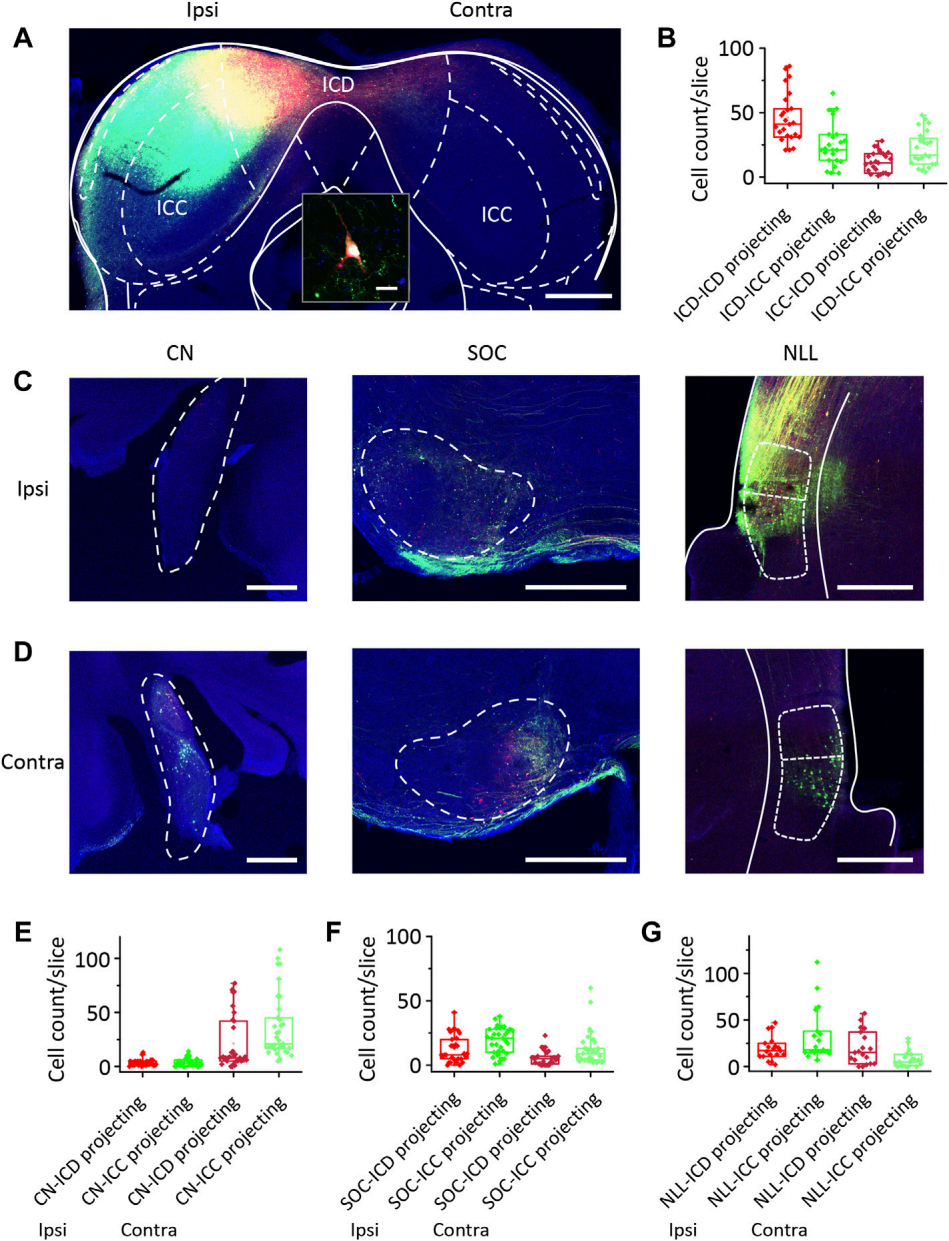
Comparison of the ICD and ICC receives projections from the contralateral ICD and the lower auditory nuclei. **(A)** Two types of retrograde viruses were injected into the left ICD and ICC and labelled neurons in the contralateral ICD and ICC. Scale bar, 500 μm. The inset shows the magnified image of a labelled neuron. Scale bar, 10 μm. **(B)** The comparison of retrograde labelled neurons number per slice in the contralateral ICD and ICC. **(C)** The retrograde labelled neurons appear in the ipsilateral cochlear nuclei (CN), superior olivary complexes (SOC) and nuclei of the lateral lemniscus (NLL). Scale bar, 500 μm. **(D)** The retrograde labelled neurons appear in the contralateral CN, SOC and NLL. **(E–G)** The comparison of retrograde labelled neurons number per slice in the ipsilateral and contralateral CN **(E)**, SOC **(F)** and NLL **(G)**. The borders of brain structures are manually outlined based on the mouse brain atlas by Paxinos and Franklin (2001) at bregma −5.02 mm (IC), −5.68 mm (CN), −5.20 mm (SOC) and −4.60 mm (NLL). The box-plots show quartiles and outliers.

Generally, auditory information ascends through the auditory pathways from the auditory nerve and crosses the midline to the contralateral side of the brain after leaving the cochlear nucleus. When determining the difference in responses to ipsilateral stimuli between the ICD and ICC, we should understand the basic pathways of the ICD and ICC. We injected retrograde AAVs encoding EGFP and mCherry into the ICC and ICD, respectively, to label commissural and ascending fibres and their cell bodies projecting to the injection sites (Figure 3A). We injected one mouse and exchanged the two viruses injected into the ICC and ICD for another mouse. For IC commissural neurons, more ICD neurons than ICC neurons projected contralaterally (Figure 3B; Table 1, ICD-ICD/ICC projecting vs. ICC-ICD/ICC projecting), and the commissural ICD neurons mainly projected to the contralateral ICD rather than the ICC (Figure 3B; Table 1, ICD-ICD projecting vs. ICC-ICD projecting). Therefore, the contralateral IC, especially the ICD, provides substantial ipsilateral information to ICD neurons. For the lower auditory nuclei, the CN, SOC and NLL sent similar projections to the ICD and ICC (Figures 3C, D), and these projections mainly came from the contralateral CN and bilateral SOC and NLL (Figures 3E–G; Table 1). In comparison, ICC-projecting neurons were more abundant and had a wider distribution in the CN and SOC than those of ICD-projecting neurons (Figures 3E–G; Table 1, CN-ICD projecting vs. CN-ICC projecting; SOC-ICD projecting vs. SOC-ICC projecting; NLL-ICD projecting vs. NLL-ICC projecting). In general, there were more projections from the ipsilateral SOC and NLL than from the contralateral side (Figures 3E, F and Table 1, ipsi SOC-ICD/ICC projecting vs. contra SOC-ICD/ICC projecting; ipsi NLL-ICD/ICC projecting vs. contra NLL-ICD/ICC projecting). However, the ICD received projections equally from ipsilateral and contralateral NLL (Figure 3F; Table 1, ipsi NLL-ICD projecting vs. contra NLL-ICD projecting). Therefore, the ICD can benefit from the contralateral NLL, which provides binaural information to the ICD that distinguishes it from that provided to the ICC. Although lower auditory nuclei project to both ICC and ICD, the pattern of bilateral projection is different between ICC and ICD. The difference in basic pathways may be the potential cause of the different responses between the ICD and ICC.

**TABLE 1:** The number of retrograde labelled neurons per slice in the inferior colliculus and lower auditory nuclei.

	ICD-Projecting		ICC-Projecting	
	Mean	Median	Mean	Median
Ipsilateral ICD-	46.21	41.5	26.04	21.5
Contralateral ICC-	12.21	11.5	22.29	20
Ipsilateral CN-	3.343	3	3.743	3
Contralateral CN-	21.31	8	35.86	21
Ipsilateral SOC-	12.75	8.5	19.34	21
Contralateral SOC-	5.688	5	12.63	9.5
Ipsilateral NLL-	19.47	17	32.63	18
Contralateral NLL-	19.74	15	8.053	5

Note: The number of labelled cells per slice is expressed as mean and median of 24 slices containing IC, 35 slices containing CN, 32 slices containing SOC, and 19 slices containing NLL, from two mice; ICD, dorsal nucleus of the inferior colliculus; ICC, central nucleus of the inferior colliculus; CN, cochlear nuclei; SOC, superior olivary complex; NLL, nucleus of the lateral lemniscus nuclei.

### Contralateral ICD Inhibition Decreases Ipsilateral Responses in Some ICD Neurons

Regarding the anatomical and morphological results, we propose that the main difference between the ICD and ICC is that the ICD accepts additional inputs from the contralateral ICD. We performed an experiment in which the contralateral ICD was inhibited by lidocaine injection to measure the effect of the contralateral ICD on ICD neurons. Before the contralateral ICD inhibition experiments, we first examined the effect of lidocaine at the injection site by recording the neurons directly affected by lidocaine to ensure that the injection system produced the desired results (Supplementary Figure S2A). The coronal slice shown in Supplementary Figure S2B illustrates the diffusion range of lidocaine (mixed with the red fluorescence dye) after the experiment. The continuous recording of neuronal spikes immediately decreased after the injection (Supplementary Figure S2C) and completely disappeared in 30 s. Then, approximately 8 min later, the firing rate gradually recovered (Supplementary Figure S2C). For the recorded population, although the recovery time varied, the inhibitory effect of lidocaine was rapid and effective, and it silenced ICD neurons for at least 5 min (Supplementary Figure S2D).

**FIGURE 4:**
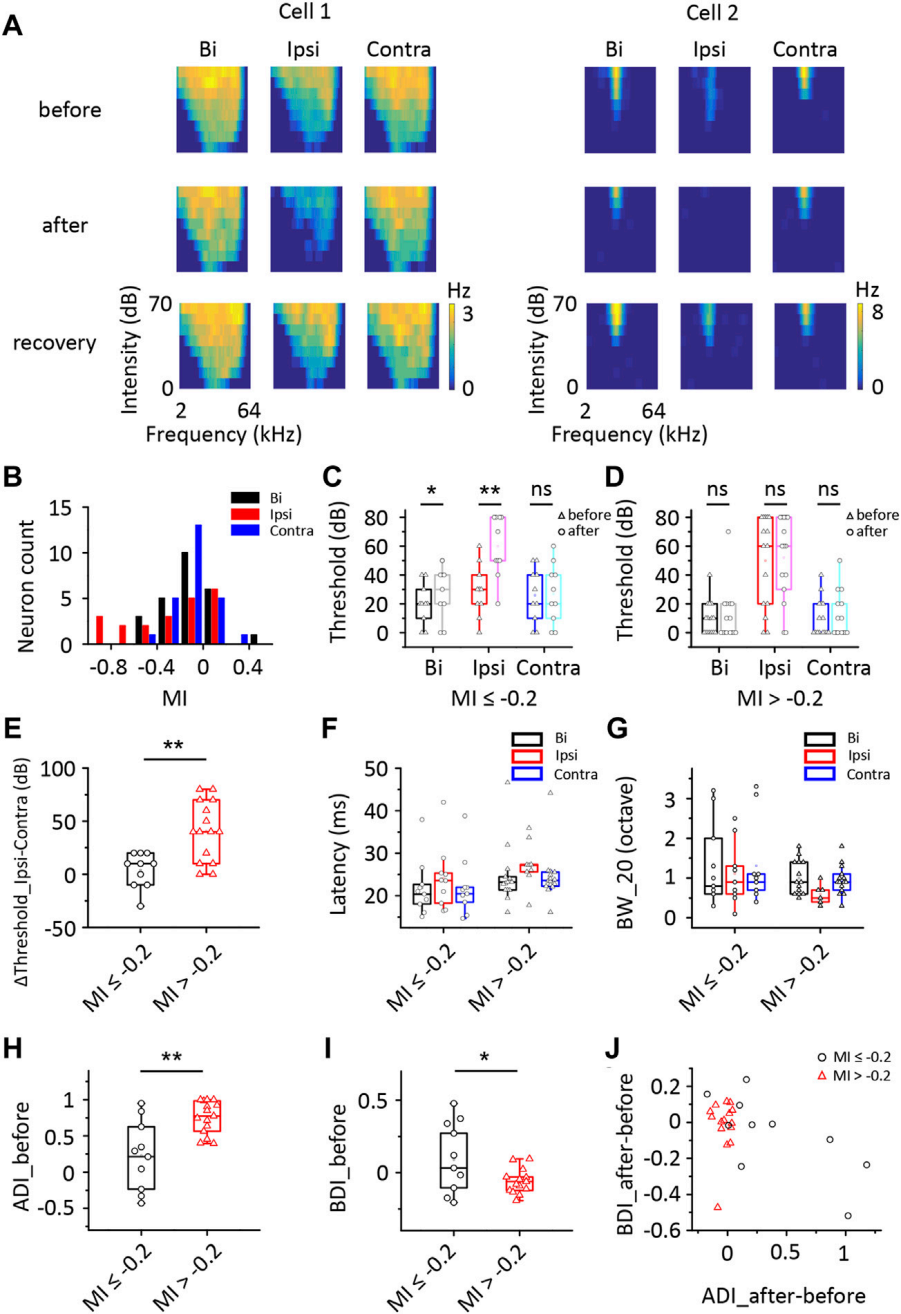
Comparison of changes in the responses of ICD neurons following the lidocaine injection. **(A)** Tonal receptive fields of two representative ICD neurons before and after the lidocaine injection and recovery from the lidocaine injection into the contralateral IC. **(B)** The modulated index (MI) distribution of the ICD neurons. Cells with an MI = −1 exhibited complete inhibition after drug treatment. **(C,D)** Changes in the minimum thresholds after the lidocaine injection were different between the two groups of ICD neurons stratified according to an MI ≤ −0.2 **(C)** or > −0.2 **(D)**. Paired *t*-test, *p < 0.05, **p < 0.001, ns, not significant. **(E–J)** Comparisons between neurons with an MI ≤ −0.2 and MI > −0.2. Comparisons of the difference in the threshold between ipsilateral and contralateral stimuli **(E)**, first spike latency **(F)**, BW_20 **(G)**, ADI **(H)**, BDI **(I)** and the change in ADI before and after injection compared with the change in BDI **(J)**. The box-plots show quartiles and outliers. Two-sample *t*-test, *p < 0.05, **p < 0.001. BW_20, bandwidth; ADI, aural dominance index; BDI, binaural dominance index.

Figure 4A shows TRFs from two representative ICD cells before injection, after injection and after recovery. The ipsilateral TRFs were markedly inhibited after contralateral ICD injection. In cell 2, the ipsilateral TRF disappeared, the responses to binaural stimuli were slightly decreased, and the responses to contralateral stimuli were almost unchanged. We defined a modulated index (MI) as the difference between entire responses before and after injection divided by their sum ([After − Before]/[After + Before]) to quantify the change in the TRF spike rate. We recorded 25 neurons from 19 mice in this experiment. Some neurons’ ipsilateral spike responses were significantly impacted by contralateral ICD silencing, while others were not (Figure 4B). Therefore, we divided the 25 neurons into two groups in which the responses to ipsilateral stimuli decreased or did not, according to the MI of the ipsilateral region of up to −0.2 (Burger and Pollak, 2001; LeBeau et al., 2001; Park et al., 2008), to compare their characteristics. For the group of neurons with MI ≤ −0.2 (*n* = 10), the average binaural and ipsilateral minimum thresholds (MTs) were higher after injection, but the contralateral MTs were not significantly different before and after the injection (Figure 4C, paired *t*-test, bi: *p* = 0.012; ipsi: *p* = 0.002; contra: *p* = 0.586; when the neuron’s MT was greater than 70 dB, we set it to 80 dB). For the group of neurons with MI > −0.2 (*n* = 15), both their binaural and monaural MTs were not significantly changed (Figure 4D, paired *t*-test, bi: *p* = 0.387; ipsi: *p* = 0.687; contra: *p* = 0.165). There were no significant differences between the two groups of neurons in the bilateral and ipsilateral MT (two-sample *t*-test, bi: *p* = 0.072, ipsi: *p* = 0.070) but there were in the contralateral MT (two-sample *t*-test, *p* = 0.020). Furthermore, we compared the difference in ipsilateral and contralateral thresholds, latency, and BW_20 between these two groups of neurons (Figures 4E–G). Significant differences in the difference between ipsilateral and contralateral thresholds were detected between these groups (Figure 4E, two-sample *t*-test, *p* = 0.001). Nevertheless, no significant differences in latency and BW_20 were observed between groups (Figures 4F, G, two-sample *t*-test, latency: bi: *p* = 0.346, ipsi: *p* = 0.297, contra: *p* = 0.354; BW_20: bi: *p* = 0.482, ipsi: *p* = 0.192, contra: *p* = 0.319). The group of neurons with MI ≤ −0.2 had a lower ADI (Figure 4H, two-sample *t*-test, *p* = 0.001) and a higher BDI (Figure 4I, two-sample *t*-test, *p* = 0.018) before the injection than those of the other groups. The distribution of the difference in ADIs after and before the injection versus the BDI difference after and before the injection indicated that the distribution remained unchanged in nearly all the neurons with MI > −0.2 after the injection (Figure 4J, one-sample *t*-test, against hypothetical mean = 0, BDI: mean = −0.016, *p* = 0.677; ADI: mean = −0.029, *p* = 0.096). In contrast, the neurons with MI ≤ −0.2 were more variable in the ADI (Figure 4J, one-sample *t*-test, against hypothetical mean = 0, BDI: mean = −0.064, *p* = 0.382; ADI: mean = 0.387, *p* = 0.028). Together, the contralateral ICD inputs may explain why some ICD neurons showed greater responses to ipsilateral stimuli and contributed to binaural summations.

### Both Excitatory and Inhibitory Synaptic Currents Decrease After Contralateral ICD Inhibition

**FIGURE 5:**
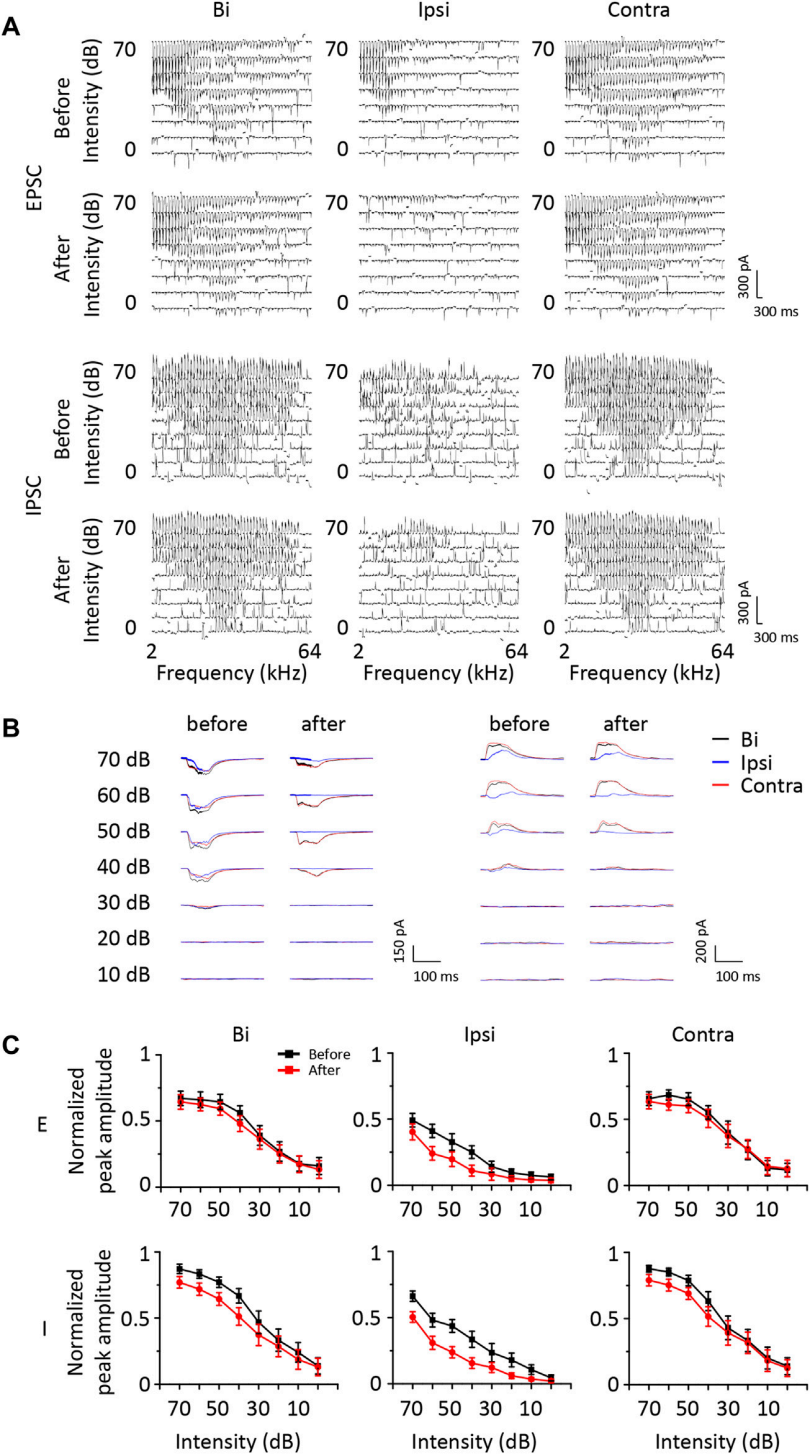
The change in synaptic responses after a contralateral ICD injection of lidocaine. **(A)** Comparison of excitatory and inhibitory synaptic tonal receptive fields of a representative ICD neuron before and after contralateral lidocaine injection and exposure to contralateral, binaural, and ipsilateral stimuli. **(B)** Comparison of synaptic responses at the BF of the same neuron shown in **(A)**. **(C)** The average change in synaptic responses in all recorded ICD neurons. Bar graphs show the means ± SEM.

**FIGURE 6:**
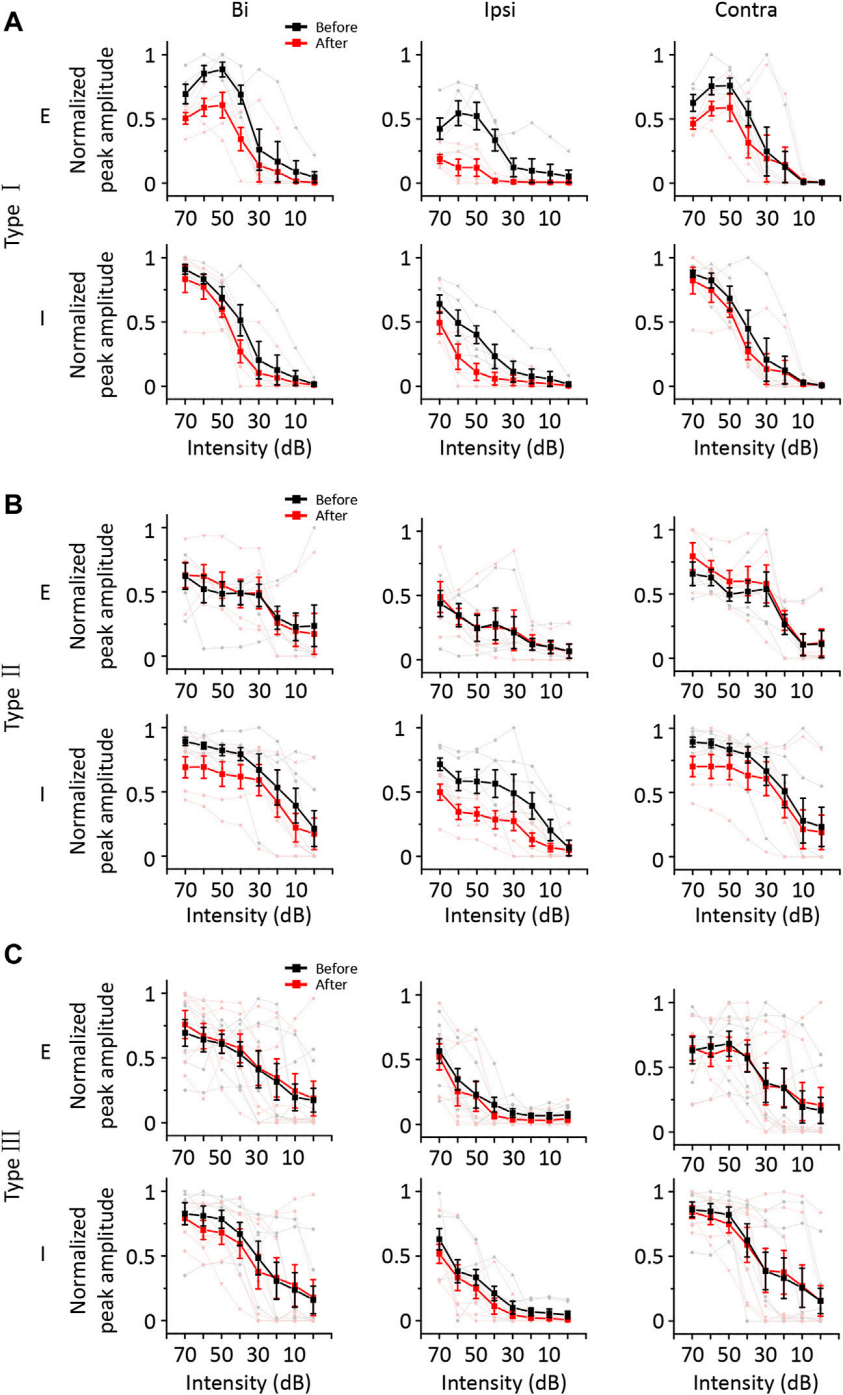
The synaptic responses of the three types of ICD neurons changed before and after the lidocaine injection and exposure to contralateral, binaural, and ipsilateral stimuli. **(A–C)** The average normalized peak amplitude of synaptic currents in the three types of ICD neurons. The three types of neurons were divided according to the change ratio of excitatory and inhibitory synaptic responses at the BF upon exposure to ipsilateral stimuli after the lidocaine injection. The excitatory currents of type I neurons decreased more significantly than the inhibitory currents **(A)**. The inhibitory currents of type II neurons decreased more significantly than the excitatory currents **(B)**. The excitatory and inhibitory currents of type III neurons were unchanged or decreased evenly **(C)**. The light lines and symbols represent the individual neurons. Bar graphs show the means ± SEM.

**FIGURE 7:**
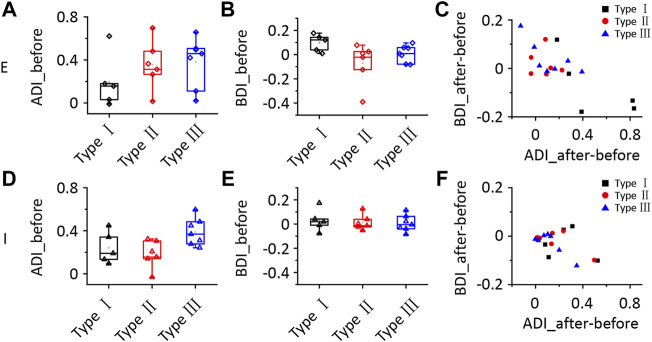
Comparison of the synaptic responses of the three types of ICD neurons following the lidocaine injection. **(A–F)** Comparison of the ADI **(A,D)**, BDI **(B,E)**, the difference in ADI before and after the lidocaine injection and the difference in BDI **(C,F)** for synaptic responses between the three types of ICD neurons. The box-plots show quartiles and outliers. ADI, aural dominance index; BDI, binaural dominance index.

According to the results described above, a group of ICD neurons integrated inputs from the contralateral ICD and added them to enhance the response to ipsilateral stimuli. However, the synaptic inputs underlying changes in ipsilateral spike responses are unclear. Hence, we performed whole-cell voltage-clamp recordings from ICD neurons with a contralateral injection of lidocaine. Excitatory and inhibitory postsynaptic currents (EPSCs and IPSCs) were dissected by clamping the cell membrane potential at −70 and 0 mV, respectively. For the representative neuron responses before injection shown in the first and third rows of Figure 5A, the ipsilateral synaptic responses were weaker than responses to binaural and contralateral stimuli in both EPSCs and IPSCs, but the shapes of ipsilateral synaptic TRFs were similar to those of responses to binaural and contralateral stimuli. As shown in the second and fourth rows of Figure 5A, the amplitude of ipsilateral synaptic responses showed a greater decrease than that observed for the response after the injection. In addition, EPSCs changed more visibly than IPSCs. We noticed that the EPSC TRFs were not the same as those of the IPSCs. The amplitudes of lower-frequency EPSC TRFs were higher, especially under ipsilateral stimulation, and the response was gradually undetectable when measured far from the BF. Since the recording of the entire whole-cell TRF is time-consuming, the experiment was not conducive to obtaining repeated recordings. Instead, we chose the BF as the stimulation to examine the neurons’ binaural and monaural synaptic responses. The representative cell shown in Figure 5B is the same neuron shown in Figure 5A, and the average EPSCs and IPSCs at BF sufficiently reflected the identical changes in TRF. Therefore, in the next experiment, we recorded the synaptic responses at a single frequency according to the BF of the excitatory currents. We successfully recorded 18 neurons from 16 mice in the injection experiment. To compare the change in amplitude between different neurons, the synaptic current data of each neuron were normalized to 0 at baseline and one at its maximum amplitude. Their normalized average amplitudes showed that both excitatory and inhibitory ipsilateral synaptic currents decreased after contralateral ICD inhibition (Figure 5C, two-way ANOVA, *F*_1,272_ (EPSC_ipsi_before_ vs. EPSC_ipsi_after_) = 16.291, *p* < 0.0001; *F*_1,272_ (IPSC_ipsi_before_ vs. IPSC_ipsi_before_) = 36.265, *p* < 0.0001). We calculated the difference in the change between EPSCs and IPSCs after the injection to further analyse all neurons. According to the differences in EPSCs and IPSCs, we divided the 18 neurons into three different types. Type I neurons exhibited a greater decrease in EPSCs than in IPSCs (Figure 6A, *n* = 5). Type II neurons displayed a greater decrease in IPSCs than in EPSCs (Figure 6B, *n* = 6). Type III neurons showed similar changes in EPSCs and IPSCs (Figure 6C, *n* = 7). As a result, type I neurons possibly exhibited a trend of decreasing ipsilateral response, type II neurons possibly exhibited a trend of increasing ipsilateral response, and the ipsilateral response of type III neurons was possibly unchanged. Their binaural responses followed the trends of ipsilateral responses. In addition, the responses to contralateral stimuli dominated the synaptic responses, as almost all ADIs (34/36) of neurons were greater than zero (Figures 7A, D, one-sample *t*-test, against hypothetical mean = 0, ADI_before for EPSC: Type I: mean = 0.196, *p* = 0.156, Type II: mean = 0.356, *p* = 0.012, Type III: mean = 0.382, *p* = 0.005; ADI_before for IPSC: Type I: mean = 0.242, *p* = 0.022, Type II: mean = 0.184, *p* = 0.018, Type III: mean = 0.389, *p* < 0.001). Regarding the BDI, the IPSCs were equal to zero, while the EPSCs of type I neurons were greater than zero (Figures 7B, E, one-sample *t*-test, against hypothetical mean = 0, BDI_before for EPSC: Type I: mean = 0.098, *p* = 0.037, Type II: mean = −0.070, *p* = 0.356, Type III: mean = 0.007, *p* = 0.799; BDI_before for IPSC: Type I: mean = 0.029, *p* = 0.516, Type II: mean = 0.013, *p* = 0.635, Type III: mean = 0.005, *p* = 0.836). A comparison of the difference in ADIs before and after injection with the difference in BDIs indicated a greater change range in EPSCs than in IPSCs (Figures 7C, F). Almost all the ADIs (32/36) of neurons were increased after the injection because of the inhibition of ipsilateral responses. The change in the BDI of neurons varied. The BDI of three type I neurons visibly decreased for EPSCs (Figure 7C). Moreover, for IPSCs, the BDI of neurons was unchanged in the majority and decreased in individual neurons (Figure 7F). As a result, we found that both excitatory and inhibitory inputs were affected by contralateral ICD inhibition; in particular, the excitatory responses to ipsilateral stimuli were significantly affected in some neurons.

### Excitation From the Contralateral ICD Contributes to Ipsilateral Responses in ICD Neurons

**FIGURE 8:**
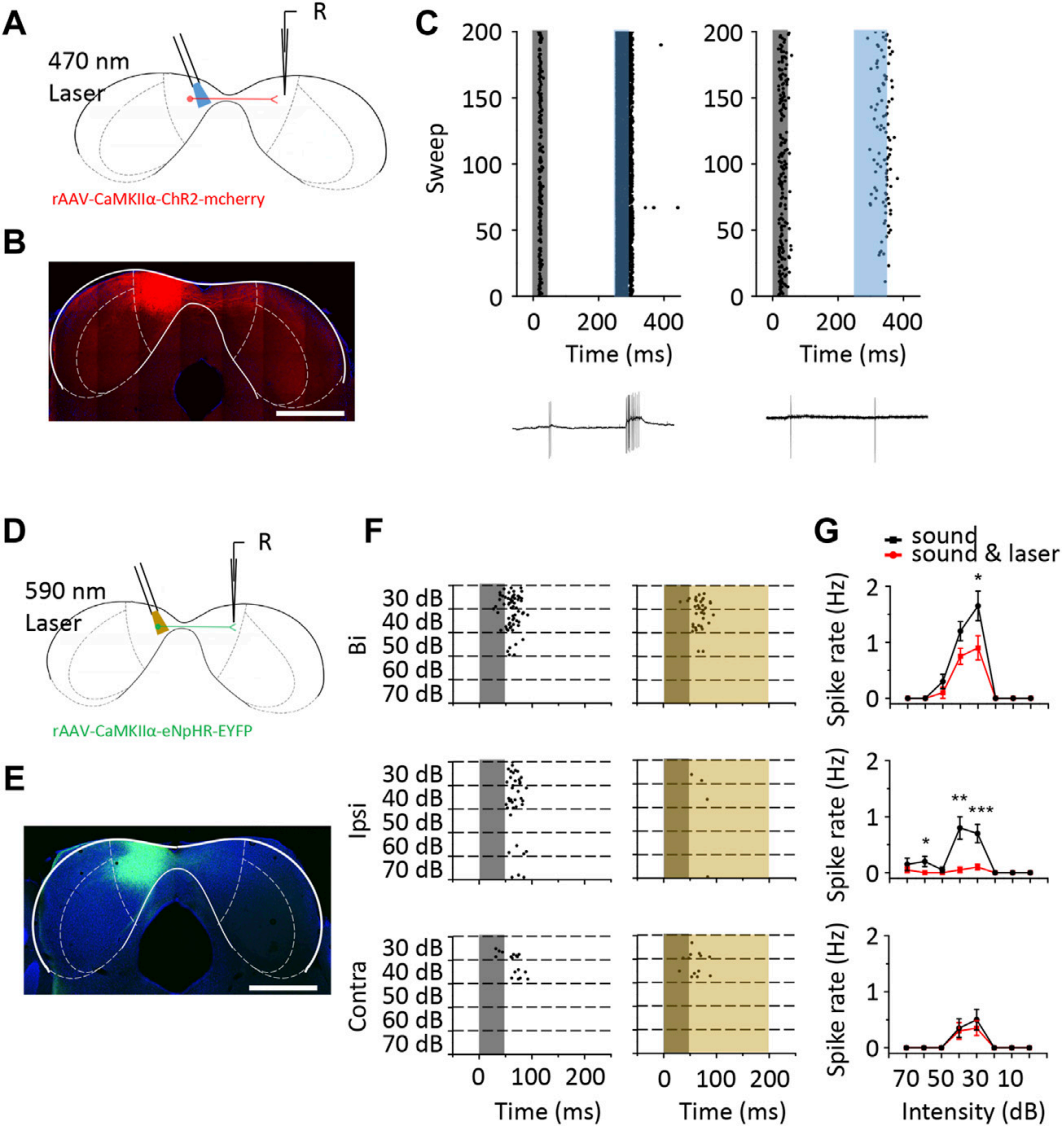
The optogenetic method revealed that the responses of ICD neurons were modulated by the contralateral ICD. **(A)** Schematic diagram of loose-patch recording from ChR2 virus-injected mouse ICD neurons. **(B)** The range of ChR2 virus infection in the ICD is shown in the coronal slice. Scale bar, 1,000 μm. **(C)** Raster plots of two representative ICD neurons that displayed an increase in the number of spikes with 470 nm laser stimulation to the contralateral ICD. The grey shaded region represents the sound stimulation. The blue shaded region represents the laser stimulation. The bottom panel shows one of the recorded traces. **(D)** Schematic diagram of loose-patch recording from eNpHR virus-injected mouse ICD neurons. **(E)** The coronal slice shows the expression of the eNpHR virus. Scale bar, 1,000 μm. **(F)** Raster plot of ICD neurons that were inhibited by 590 nm laser stimulation of the contralateral ICD. The yellow shaded region represents the laser stimulation. **(G)** Comparison of the rate-intensity function between laser on and off responses to binaural (top panel), ipsilateral (middle panel) and contralateral (bottom panel) sound stimulation. The borders of IC subdivisions are manually outlined based on the mouse brain atlas by Paxinos and Franklin (2001) at bregma −5.02 mm. Bars show the means ± SEM. Two-sample *t*-test, *p < 0.05, **p < 0.001, ***p < 0.0001.

We next examined the contribution of excitatory neurons in the contralateral ICD to ipsilateral responses using the optogenetic technique. We injected CaMKIIα-ChR2 and CaMKIIα-eNpHR viruses into the left ICD of four mice, respectively. Three weeks after the CaMKIIα-ChR2 virus injection, the contralateral ICD was subjected to loose-patch recording (Figures 8A, B). During the electrophysiological recordings, we first tested ipsilateral responses of each cell and then administered laser light stimulation at the injection site. Here, we recorded from neurons that exhibited spikes evoked by 470 nm light stimulation (Figure 8C). This finding indicated that excitatory neuron projections could provide excitation through monosynaptic or polysynaptic pathway to ICD neurons. Moreover, we also recorded a neuron that was inhibited by light stimulation, especially for responses to bilateral and contralateral sound stimuli (Supplementary Figure S3). This inhibition can be explained by the activation of local interneurons. These findings are consistent with the whole-cell recordings, as both excitatory and inhibitory inputs were potentially affected by contralateral ICD silencing. Furthermore, we used the CaMKIIα-eNpHR virus to inhibit contralateral neurons (Figures 8D, E). We first recorded data from neurons at the injection site to ensure the inhibitory effect of the virus. As the duration of light stimulation increased, the sound-evoked spikes of the infected neuron were completely inhibited (Supplementary Figure S4). We then recorded from neurons at the contralateral ICD and set the duration of light stimulation as 200 m. A representative neuron showed that the binaural and ipsilateral spike responses were inhibited by 590 nm light stimulation (Figures 8F, G). Based on these findings, excitation from the contralateral ICD contributes to the ipsilateral responses in neurons.

## Discussion

In this study, we compared the differences in binaural responses between the ICD and ICC using loose-patch recordings and found that the binaural responses were different in the ICD. By combining drug injections and electrophysiological recordings, we confirmed that the contralateral ICD provides excitatory inputs to support the responses to ipsilateral stimuli. These observations suggest that in some ICD neurons, especially ipsilateral highly reactive neurons, their excitatory inputs may be derived from the contralateral IC, which also sums the binaural responses.

According to cytoarchitectonics, the IC is mainly divided into three subdivisions: the central nucleus, the external cortex (ICX) and the dorsal cortex (Morest and Oliver, 1984; Meininger et al., 1986; Malmierca et al., 1995). The ICC has a good tonotopic organization and is considered to have a more typical tone-evoked neuronal response than the ICD and ICX, with short latencies, sharp tuning and robust tonic firing. Since the precise borders of the different nuclei of the IC have been disputed based on different classifications (Meininger et al., 1986; Coote and Rees, 2008; Grana et al., 2017; Gay et al., 2018), the ICD and ICX neurons have been less examined than ICC neurons in past studies. However, recent studies using two-photo imaging have greatly expanded the knowledge of the physiological properties of the dorsal surface of the IC (Geis et al., 2011; Geis and Borst, 2013a; Geis and Borst, 2013b; Ito et al., 2014; Barnstedt et al., 2015; Chen and Song, 2019; Wong and Borst, 2019; Wang et al., 2021). More detailed spatial tonotopic gradients and electrophysiological properties were confirmed in the dorsal IC, refuting claims that the ICD lacks topographic organization or is simply an extension of the ICC (Willott, 2001; Malmierca et al., 2008; Lumani and Zhang, 2010; Barnstedt et al., 2015; Wong and Borst, 2019). This finding also inspired us to study the difference in binaural responses between ICD and ICC neurons. Our study focused on the binaural properties of neurons in the ICD and their function in auditory processing. Unlike previous studies, which used open field sounds or contralateral sounds and optionally recorded ICD neurons when electrodes were placed at sites along the pathway to the ICC, we used the closed sound field to record responses to both ipsilateral and contralateral stimuli and investigated a large sample of neurons based on systematic control of the recording region (although locations did not cover the whole region, the selections were representative). Therefore, our results provide more detailed information and are not completely identical to those of other studies. In our observations, the differences in latency (Figure 2C) and width of the receptive field (Figure 2B) were consistent with those of previous reports and indicated different pathways to the ICD and ICC (Syka et al., 2000; Lumani and Zhang, 2010; Barnstedt et al., 2015). In addition, the ipsilateral BW was narrower, which indicated contralateral dominance (Figure 2B). The ipsilateral TRF in the ICD did not match the contralateral TRF well in comparison to those of the ICC (Figure 2A). However, the most obvious difference is the response to ipsilateral stimuli, in which ICD neurons show larger responses and a lower threshold (Figures 2D–I). In other words, a higher percentage of ICD neurons displayed stronger responses to ipsilateral stimuli than ICC neurons. As a result, the ADI of the ICD is less than that of the ICC. In previous papers, the binaural property was described as EE, EI and EO neurons (Semple and Aitkin, 1979; Semple and Kitzes, 1987; Brückner and Rübsamen, 1995). In contrast, EI neurons, which receive excitatory contralateral inputs and inhibitory ipsilateral inputs and exhibit a larger ADI, made up the largest proportion of neurons in these reports (Semple and Aitkin, 1979; Irvine and Gago, 1990; Mendelson and Grasse, 1992; Brückner and Rübsamen, 1995; Lu and Jen, 2003; Mei et al., 2013; Xiong et al., 2013), which may not occur in the ICD.

Although neurons sensitive to contralateral ICD inhibition showed smaller differences in the responses between ipsilateral and contralateral stimuli (ΔThreshold_Ipsi−Contra∼30 dB, ADI∼1, Figures 4E, H), the reverse is not always true: Not all neurons with smaller differences in the responses between ipsilateral and contralateral stimuli were affected by the inhibition (Figure 4J). According to morphological findings via retrograde virus tracing (Figure 3), in addition to the contralateral inferior colliculus, the lower nuclei, ipsilateral CN, SOC, and NLL may provide ipsilateral stimuli and binaural integration information. The contralateral NLL, in particular, has more projections to the ICD than the ICC and is generally thought to provide IC inhibitory inputs. Whether the NLL plays a role in the discrepancies between the ICD and ICC binaural responses remains to be explored. Moreover, based on our observations, the ICC and ICD also receive substantial cortical feedback projections (Supplementary Figure S5), while auditory responses in the auditory cortex are likely strongly suppressed under anaesthesia (Wang et al., 2005; Alkire et al., 2008; Harris et al., 2011; Barnstedt et al., 2015). This finding is consistent with previous reports that neurons in the ICD and dorsal ICC receive extensive innervation from a combination of ascending and descending auditory inputs (Chen and Song, 2019; Wong and Borst, 2019). Thus, we cannot rule out the possibility that the auditory cortex can provide binaural information to the ICD while awake, which should be further studied in the future.

Although the injection of lidocaine is a method widely used in recent studies, the precision and specificity of the drug are limited. Regarding the ipsilateral recordings after the injection, some neurons showed insufficient recovery (Supplementary Figure S2D). The effect of drug remnants or delayed side effects were considered. We performed a control experiment using optogenetic to verify the effect of contralateral ICD inhibition. CaMKII was thought to be expressed specifically in non-GABA neurons, including the IC (Benson et al., 1992; Liu and Murray, 2012). In the cerebral cortex, the CaMKII promotor was employed to label glutamatergic neurons, while the promotor was not examined in the IC. Although in a small amount of data, the contralateral ICD was verified to supplement the ipsilateral responses of some ICD neurons (Figure 8). This finding may explain the difference in the responses to ipsilateral stimuli between the ICD and ICC. Meanwhile, drug injection may affect transmission fibres from other regions. Thus, the percentage of neurons with ipsilateral responses decreased to a lesser extent than that with light inhibition in our experiments. Moreover, optogenetic virus infection may be incomplete and the light-stimulated region or light intensity might be limited. The synaptic responses were not the same as those from the extracellular recordings in terms of the distribution of neuron types (Figures 4–7). Generally, synaptic changes would likely be more sensitive (Liu et al., 2019; Wang et al., 2019; Wu et al., 2021). Therefore, some subthreshold modulation was not reflected in spikes. In addition, the different time courses of excitatory and inhibitory synaptic currents would generate different spike outcomes. In our study, the lack of evidence in membrane potential made direct comparison of neuron types inappropriate (He et al., 2017). Both the loose-patch and whole-cell recordings displayed a change in the responses to contralateral stimuli after the injection. We were unable to exclude the possibility that lidocaine diffused to the contralateral ICD or that rundown occurred in the whole-cell recording. Nevertheless, the interaction of bilateral ICDs may be a more suitable explanation.

The intercollicular commissure between ICs has not been completely clearly defined. Early immunohistochemical studies showed that there was a large proportion of excitatory commissural fibres in the ICs (González-Hernández et al., 1996; Hernández et al., 2006). Nevertheless, a much higher level of inhibition than expected was observed in previous *in vitro* studies (Smith, 1992; Moore et al., 1998). Some of this inhibition might be caused by excitatory neurons activating local interneurons. Generally, because of the existence of numerous inhibitory interneurons, there is a higher proportion of inhibitory interactions than excitatory interactions (Mei et al., 2013). Some papers have reported that the number of neurons inhibited by contralateral neurons was greater than the number of neurons facilitated (Jen et al., 2002; Wu and Jen, 2008; Mei et al., 2016). However, the proportion of facilitated neurons was reported to be higher than that of inhibited neurons for non-V-shaped TRF neurons (Orton and Rees, 2014). Although these studies were focused on the ICC, their findings were similar to our results in the ICD showing that both inhibition and facilitation arise from contralateral areas. The difference between the ICD and the ICC in bilateral interactions may be that the ICD contains more facilitated neurons. Moreover, contralateral excitation exerts a direct or indirect effect according to the transmission time and can even activate interneurons to lead to inhibition. Direct projection always provides stronger inputs (Figure 8C, left panel). Another virus tracing study revealed that disinhibition pathways could be found in abundance in the intercollicular commissures between ICDs (Chen et al., 2018). We regret that we were unable to determine how the composition and proportion of excitatory and inhibitory fibres or commissural fibres to the ICD, the role of the interneurons, and their distribution in this study. All these parameters require further investigation.

The robust responses of ipsilateral ICD neurons indicated that the supplemental excitatory input from the contralateral ICD enhanced the fundamental reaction. Moreover, contralateral supplementary input also modulates binaural responses. The BDI indicated that the contralateral inputs contributed to binaural integration in some ICD neurons. In addition, the ipsilateral responses of some ICD neurons were integrated at the lower auditory nuclei as their BDIs were unchanged after contralateral ICD inhibition. The role of the response to ipsilateral stimuli may contribute to integrating binaural responses, such as increasing the responses at the central frequency or intensity and decreasing the responses at frequencies around the centre. This process may provide a clearer discrimination to facilitate binaural hearing.

In conclusion, some ICD neurons receive excitatory inputs from the contralateral ICD, which enhance their responses to ipsilateral stimuli and modulate responses to binaural stimuli, and these neurons have the characteristics of a low minimum threshold for ipsilateral stimuli or a small difference in the minimum threshold between ipsilateral and contralateral stimuli.

## Data Availability

The original contributions presented in the study are included in the article/Supplementary Material, further inquiries can be directed to the corresponding author.
