# Supplementary material for: Bilateral Interactions in the Mouse Dorsal Inferior Colliculus Enhance the Ipsilateral Neuronal Responses and Binaural Hearing
Source: Front Physiol. 2022 Apr 19;13:854077. doi: 10.3389/fphys.2022.854077 (PMC9061965; doi:10.3389/fphys.2022.854077)
Supplement: Supplementary file 1 [file Presentation1.PDF]

Supplementary Figures

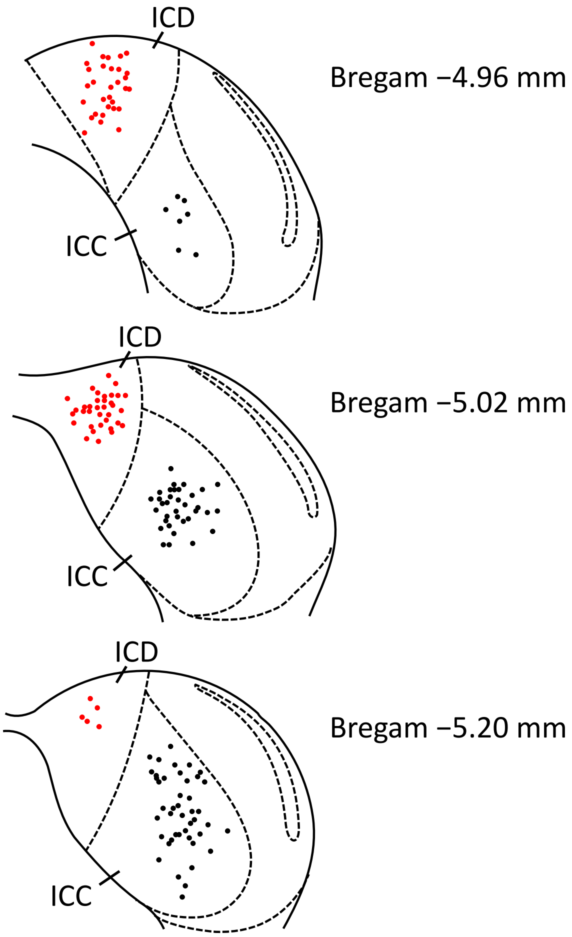

Supplementary Figure 1. Schematic drawings based on the mouse brain atlas by Paxinos and Franklin (2001) show the reconstructed recording sites in the ICD (red, n = 69 cells) and ICC (black, n = 80 cells).

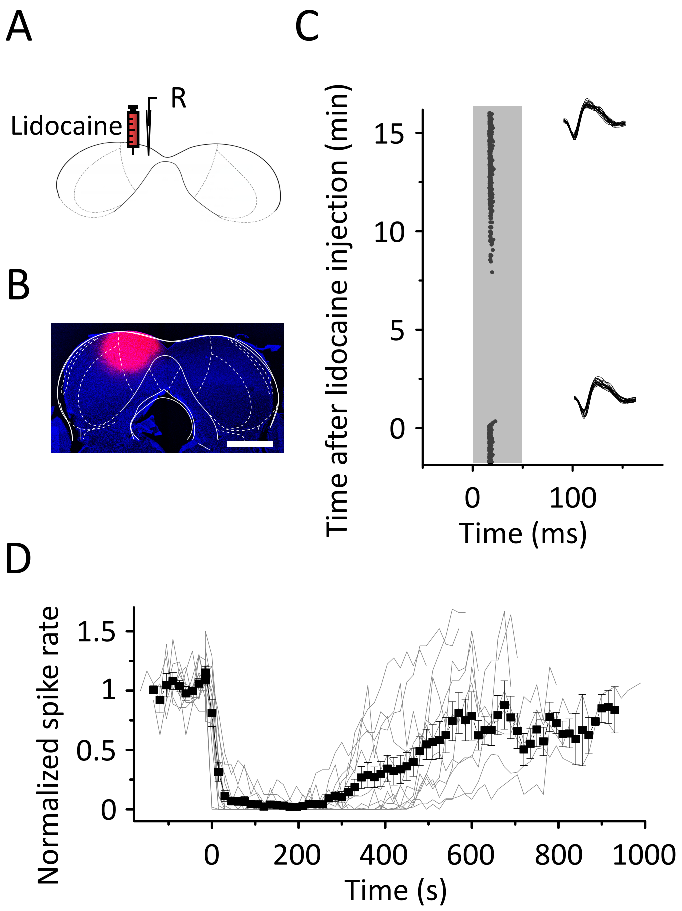

Supplementary Figure 2. The microinjection of lidocaine suppresses the spike responses of local ICD neurons. **(A)** Schematic diagram of the lidocaine microinjection model. R indicates the recording electrode. **(B)** The diffusion range of the lidocaine microinjection. The borders of IC subdivisions are manually outlined based on the mouse brain atlas by Paxinos and Franklin (2001) at bregma −5.02 mm. Scale bar, 1000 µm. **(C)** Raster plot of changes in the spike response (black dots) of the representative neuron with the injection of lidocaine. The shaded region represents the duration of the tone stimulation. The spike shapes of neuronal activity before (lower) and after (upper) injection are shown in the insets. **(D)** The average normalized spike rate of the neurons was inhibited by the local lidocaine injection. The solid symbols represent means ± SEM. The grey lines represent individual neurons.

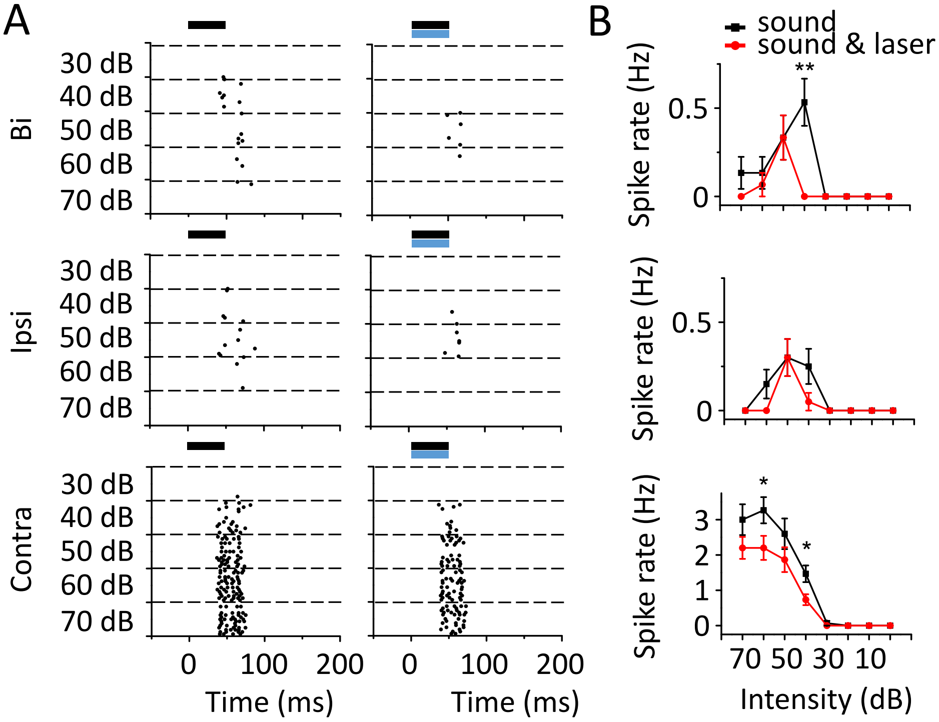

Supplementary Figure 3. The ICD neuron was inhibited by the blue laser stimulus to the ChR2 virus-infected contralateral ICD. **(A)** Raster plot of ICD neurons that were inhibited by 470 nm light stimulation of the contralateral ICD. The black and blue bars represent the duration of the tone stimulation and laser stimulation, respectively. **(B)** Comparison of the rate-intensity function between light on and -off responses to binaural (top panel), ipsilateral (middle panel) and contralateral (bottom panel) sound stimuli. Bars represent means ± SEM. Two-sample *t*-test, *$p < 0.05$, **$p < 0.001$.

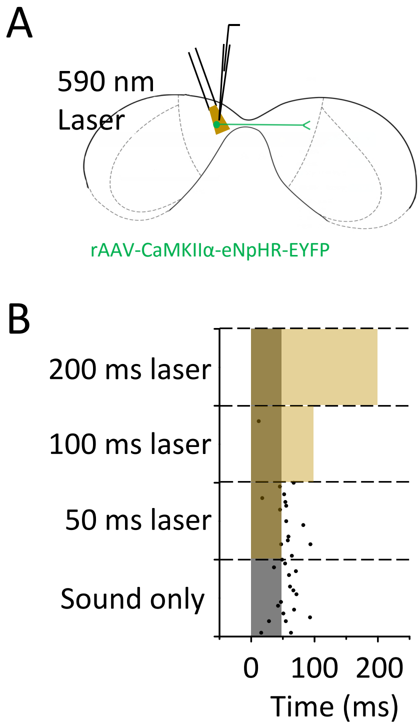

Supplementary Figure 4. The eNpHR virus-injected ICD neuron was inhibited by the yellow laser stimulus directly. **(A)** Schematic diagram of loose-patch recording in eNpHR virus-injected mouse ICD neurons with 590 nm laser stimulus. **(B)** Prolonging the duration of 590 nm light exposure generated stable inhibition of sound-evoked responses in ICD neurons on the injected side. The grey shaded region represents the duration of the tone stimulation. The yellow shaded region represents the duration of the laser stimulation.

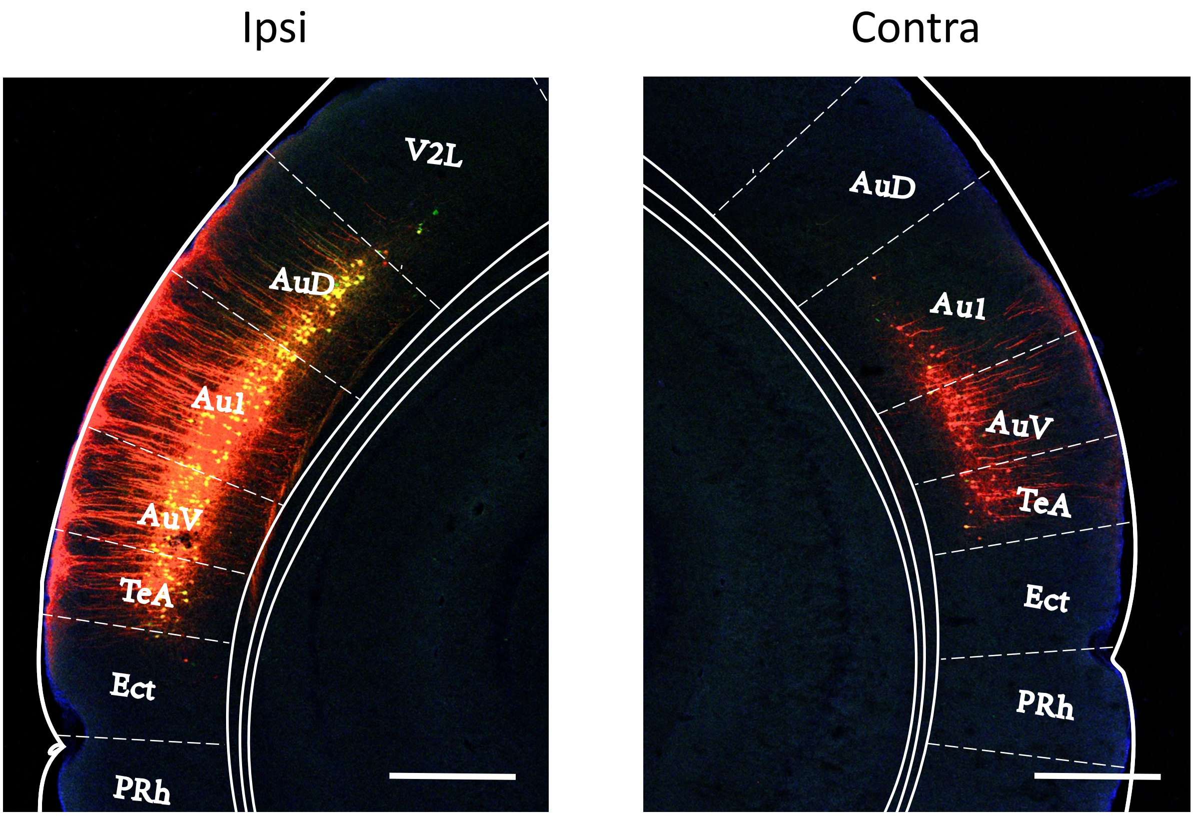

Supplementary Figure 5. The retrograde labeled neurons appear in the ipsilateral and contralateral auditory cortex after retrograde tracing viruses were injected into the ICD (red) and ICC (green). The borders of brain structures are manually outlined based on the mouse brain atlas by Paxinos and Franklin (2001) at bregma −3.28 mm. Scale bar, 500 µm.
